# Identification of a single amino acid in GluN1 that is critical for glycine-primed internalization of NMDA receptors

**DOI:** 10.1186/1756-6606-6-36

**Published:** 2013-08-13

**Authors:** Lu Han, Verónica A Campanucci, James Cooke, Michael W Salter

**Affiliations:** 1 Program in Neurosciences & Mental Health, Hospital for Sick Children, 555 University Ave, Toronto, Ontario M5G 1X8, Canada; 2 Department of Physiology, University of Toronto, Toronto, Ontario M5G 1X8, Canada; 3 Neural Systems and Plasticity Group, Department of Physiology, University of Saskatchewan, Saskatoon, Saskatchewan S7N 5E5, Canada

**Keywords:** NMDA Receptors, Glycine, Internalization, Endocytosis, Dynamin, GluN1, GluN2

## Abstract

**Background:**

NMDA receptors are ligand-gated ion channels with essential roles in glutamatergic synaptic transmission and plasticity in the CNS. As co-receptors for glutamate and glycine, gating of the NMDA receptor/channel pore requires agonist binding to the glycine sites, as well as to the glutamate sites, on the ligand-binding domains of the receptor. In addition to channel gating, glycine has been found to prime NMDA receptors for internalization upon subsequent stimulation of glutamate and glycine sites.

**Results:**

Here we address the key issue of identifying molecular determinants in the glycine-binding subunit, GluN1, that are essential for priming of NMDA receptors. We found that glycine treatment of wild-type NMDA receptors led to recruitment of the adaptor protein 2 (AP-2), and subsequent internalization after activating the receptors by NMDA plus glycine. However, with a glycine-binding mutant of GluN1 – N710R/Y711R/E712A/A714L – we found that treating with glycine did not promote recruitment of AP-2 nor were glycine-treated receptors internalized when subsequently activated with NMDA plus glycine. Likewise, GluN1 carrying a single point mutation – A714L – did not prime upon glycine treatment. Importantly, both of the mutant receptors were functional, as stimulating with NMDA plus glycine evoked inward currents.

**Conclusions:**

Thus, we have identified a single amino acid in GluN1 that is critical for priming of NMDA receptors by glycine. Moreover, we have demonstrated the principle that while NMDA receptor gating and priming share a common requirement for glycine binding, the molecular constraints in GluN1 for gating are distinct from those for priming.

## Introduction

NMDA receptors (NMDARs) constitute a major subtype of glutamate receptor and play important roles in numerous physiological and pathophysiological processes in the CNS [1]. NMDARs are unique in the glutamate receptor family in that they require a co-agonist, glycine, in addition to glutamate in order to gate receptor opening [2]. The core of NMDARs is a heterotetrameric assembly of two GluN1 and two GluN2 subunits; glycine binds to GluN1 and glutamate to GluN2 [3]. NMDAR heterotetramers assemble in the endoplasmic reticulum and, after processing in the Golgi, mature NMDARs are trafficked to the cell surface to synaptic, as well as to extrasynaptic sites.

The number of cell-surface NMDARs is critically regulated by endocytosis [4] which is either constitutive or regulated, i.e. induced by stimulation. Both constitutive and regulated NMDAR endocytosis are dynamin-dependent [5,6]. Regulated NMDAR endocytosis may be evoked either heterologously, by stimulation of other receptors such as group1 metabotropic glutamate receptors [7] or alpha-7 nicotinic receptors [8], or homologously, by direct co-agonist stimulation of the NMDARs themselves [6,9].

NMDARs can be ‘primed’ for homologous endocytosis by selectively stimulating the receptors with glycine [10]. However, glycine stimulation alone does not induce internalization of NMDARs. Rather the primed receptors are internalized upon subsequent stimulation with glutamate and glycine. Thus, glycine readies the receptors, so they can be internalized when activated by both co-agonists. At glutamatergic synapses the glycine transporter, GLYT1 [11], normally maintains extracellular glycine concentration at a level below that required to induce priming. Blocking GLYT1 activity sufficiently produces depression of NMDAR-mediated synaptic responses and limits NMDAR-dependent plasticity [12]. Thus, glycine-primed internalization may have a critical role under conditions where endogenous glycine levels rise such as high levels of neuronal firing or CNS damage by hypoxia or trauma [13-16].

As a molecular correlate of priming, glycine stimulation causes the AP-2 endocytic adaptor complex to be recruited to NMDARs [10]. Thus, a working model is that there are two mechanistically separable steps: priming with AP-2 recruitment caused by glycine alone and endocytosis *per se* caused by glutamate and glycine co-stimulation [6]. In the present study we tested an implicit assumption that the glycine priming process is mediated through GluN1. We carried out our studies using wild-type and mutant NMDARs expressed heterologously. First, we established with wild-type receptors that glycine primes internalization of recombinant NMDARs, fully recapitulating the characteristics of glycine-primed internalization of native NMDARs. Subsequently, we found that mutations in GluN1 prevented priming of NMDARs by glycine, and we discovered that a single amino acid, A714, is critical for glycine priming.

## Results

To investigate molecular determinants for glycine-primed internalization of NMDARs we expressed wild-type or mutant GluN1/GluN2A or GluN1/GluN2B receptors in HEK293 cells. We used four different approaches to study priming and internalization of NMDARs: i) whole-cell recording of NMDAR currents, ii) NMDAR surface expression using cell ELISA, iii) fluorescence imaging of internalization of NMDARs and iv) co-immunoprecipitation of NMDARs with the AP-2 complex.

### Glycine-primed internalization of wild-type NMDARs

**Figure 1:**
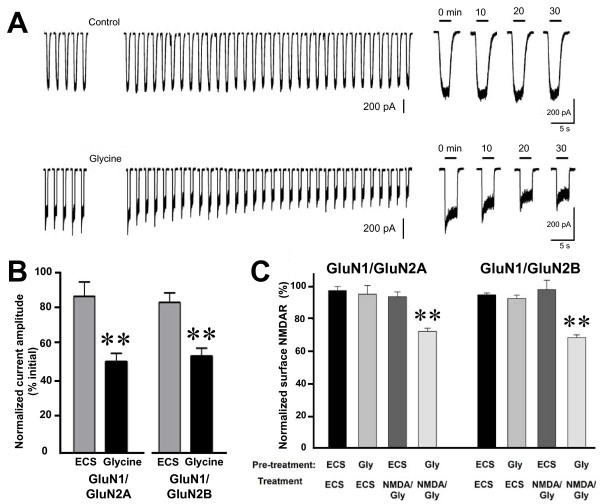
**Glycine treatment primes depression of wild type recombinant NMDA receptors mediated currents. A**, Representative traces show responses to the test applications recorded from cells expressing GluN1/GluN2A that were treated with ECS (upper) or Glycine (lower). Glycine (100 μM) was applied for 5 min, where indicated. For the expanded traces on the right, time indicates time after glycine treatment. **B**, Histogram showing average normalized peak NMDA currents evoked by NMDA/glycine test applications 20 min after conditioning with glycine (100 μM) to both GluN2A and GluN2B expressing HEK293 cells. **C**, NMDAR internalization (mean ± s.e.m.; percentage of total) measured by cell ELISA assay in HEK293 cells expressing wild type recombinant NMDAR. Cultures (n = 6) were pre-treated with ECS or ECS containing glycine (100 μM) plus APV (100 μM) followed by ECS or with NMDA (50 μM) plus glycine (1μM). ** indicates p < 0.01 compared with ECS control.

With wild-type NMDARs, we found that after treating cells with glycine (100 μM; 5 min) the amplitude of NMDAR-mediated currents – evoked by test applications of NMDA (50 μM) plus glycine (1 μM) – was reduced significantly as compared with cells not treated with glycine (Figure 1A and B). Twenty min after the end of glycine application the NMDAR currents were: 53 ± 5% (p < 0.01) of baseline for GluN1/GluN2A receptors and 57 ± 5% (p < 0.01) of baseline for GluN1/GluN2B receptors. NMDAR current amplitude remained stable at the depressed levels for up to 1 hr after glycine treatment (not illustrated). Thus, with either wild-type GluN1/GluN2A or wild-type GluN1/GluN2B recombinant receptors glycine reliably and reproducibly primed NMDARs currents for depression.

To investigate NMDAR cell-surface expression, we labeled NMDARs under non-permeabilizing conditions using an antibody directed against an extracellular epitope on GluN1, and measured the cell-surface level by ELISA. We found that NMDAR cell-surface level was stable when the cells were treated with ECS alone (Figure 1C). Moreover, NMDAR cell-surface level did not change for cells pre-treated with ECS and then treated with NMDA (50 μM) plus glycine (1 μM), i.e. concentrations equal to those of the test application of NMDA plus glycine used in the electrophysiological experiments. NMDAR cell surface level was also unchanged by pre-treating the cells with glycine (100 μM) and then treating with ECS. By contrast, NMDAR cell-surface level was significantly decreased by pre-treating the cells with glycine (100 μM) and treating with NMDA (50 μM) plus glycine (1 μM) (Figure 1C): surface GluN1/GluN2A receptor levels were reduced to 72 ± 2% (p < 0.01) of control and surface GluN1/GluN2B receptors decreased to 68 ± 2% (p < 0.01). Thus, the level of wild-type GluN1/GluN2A or GluN1/GluN2B receptors on the cell surface was reduced by glycine pre-treatment followed by NMDAR activation with NMDA plus glycine.

**Figure 2:**
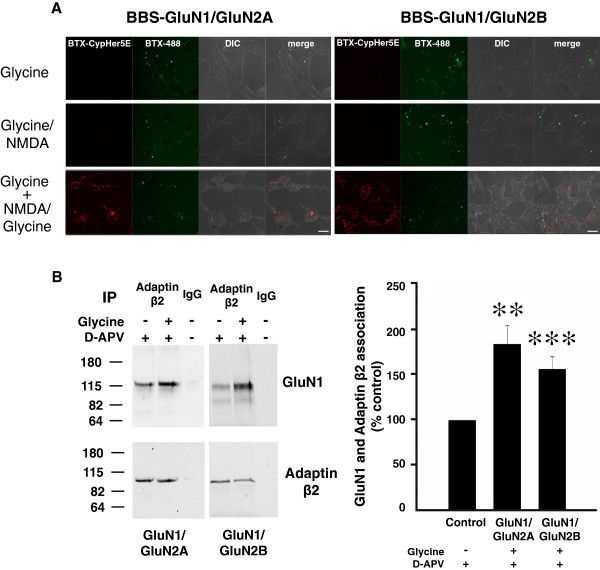
**Glycine treatment primes internalization and recruitment of adaptin β2 to the wild type recombinant NMDA receptors. A**, Fluorescence images of HEK 293 cells expressing GluN2A and GluN2B containing NMDARs labeled with CypHer5E-conjugated BTX and BTX-AF488 treated with NMDA (50 μM) plus glycine (1μM) with or without glycine pre-treatment. Scale bar = 10 μm. **B**, *Left* Representative Western blot of co-immunoprecipitation of NMDARs with adaptin β2 from cell lysate treated with glycine (100μM) plus APV (100μM) or APV (100μM) alone. *Right* Quantification is summarized in the histogram showing mean GluN1-adaptin β2 association after glycine treatment (n = 10). Data are presented as mean percent (± s.e.m.) of APV treated group. Data were normalized to the amount of adaptin β2 immunoprecipitated. ** indicates p < 0.01 *** indicates p < 0.001 compared to control.

To visualize changes in NMDAR localization we took advantage of the fluorochrome CypHer5E which is fluorescent in acidic pH, such as in endosomes, but which is non-fluorescent at neutral or basic pH [17,18]. CypHer5E was conjugated to α-bungarotoxin (BTX-CypHer5E), and we engineered a 13-amino acid BTX-binding sequence (BBS) [19-21] at the N-terminus of the GluN1 subunit. Currents evoked through the BBS-GluN1/GluN2A or BBS-GluN1/GluN2B receptors were indistinguishable from those of wild-type receptors, as was glycine-primed reduction of BBS-NMDAR currents (data not shown). At the start of each imaging experiment, we tagged BBS-NMDARs on the cell surface with BTX-CypHer5E at 4°C to prevent constitutive internalization. After treatment, the BBS-NMDARs remaining on the cell surface were labeled with BTX-conjugated Alexa Fluor 488 (BTX-AF488). In cells expressing BBS-GluN1/GluN2A or BBS-GluN1/GluN2B receptors, we observed robust Alexa Fluor 488 signal indicating expression of the BBS-NMDARs. In cells expressing BBS-NMDARs, we saw no CypHer5E signal above background after treating with glycine (100 μM) or with NMDA (50 μM) plus glycine (1 μM) (Figure 2A). By contrast, in cells pre-treated with glycine (100 μM) followed by NMDA (50 μM) plus glycine (1 μM) we observed bright red punctate CypHer5E fluorescence (Figure 2A). CypHer5E puncta were seen with BBS-GluN1/GluN2A receptors and with BBS-GluN1/GluN2B receptors. Some overlap of the CypHer5E and BTX-AF488 signals was observed, due primarily to signals in different planes being superimposed in the projection images. The CypHer5E punctate signal was lost upon intracellular alkalinization (not illustrated) indicating that BBS-NMDARs that had been on the cell surface at the start of the experiment were in an acidic intracellular compartment at the end of the experiment. We take these findings as evidence that glycine pre-treatment followed by NMDAR activation with NMDA plus glycine causes internalization of either GluN1/GluN2A or GluN1/GluN2B receptors.

A molecular signature of glycine priming is recruitment of the AP-2 adaptor complex to native NMDARs in hippocampal neurons [10]. To determine whether glycine stimulation recruits AP-2 to recombinant NMDARs, we examined the association of GluN1/GluN2A or GluN1/GluN2B receptors with the adaptin β2 subunit of endogenous AP-2 in the HEK cells. In cells treated with ECS alone, we detected a basal association of NMDARs and AP-2 by co-immunoprecipitation of GluN1 with an antibody against adaptin β2 but not with a non-specific IgG (Figure 2B). After stimulating with glycine (100 μM) the amount of GluN1 that co-immunoprecipitated with anti-adaptin β2 increased significantly with GluN1/GluN2A or with GluN1/GluN2B receptors (Figure 2B); there was no alteration of adaptin β2 immunoprecipitated. As D-APV was always included together with the glycine treatment we examined whether D-APV might contribute to the enhanced association of GluN1 and adaptin β2. However, we found that treating with D-APV (100 μM) alone produced no significant change in the amount of GluN1 co-immunoprecipitated by anti-adaptin β2 (Additional file 1: Figure S1). Therefore, glycine stimulation increased the association of recombinant NMDARs with AP-2.

**Figure 3:**
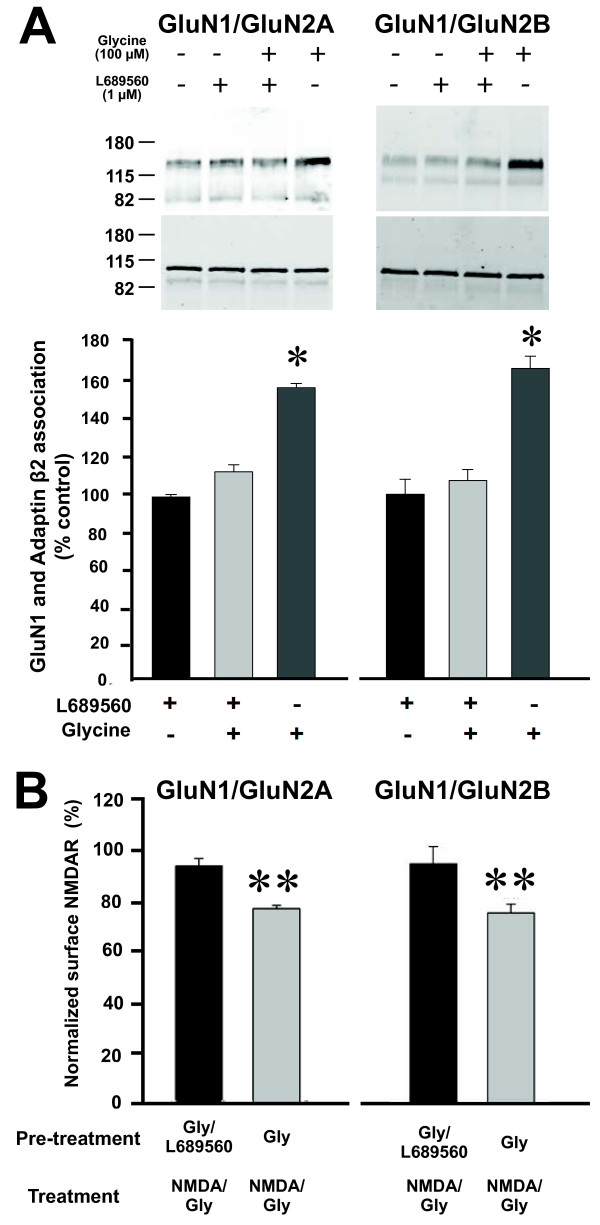
**Pharmacological blocker of the glycine binding site prevents glycine primed NMDAR endocytosis. A**, *Top* Representative Western blot of co-immunoprecipitation of NMDARs with adaptin β2 from HEK293 cells expressing recombinant wild type NMDARs treated with ECS, glycine (100 μM) with or without L689560 (1μM), or with L689560 alone. *Bottom*, Histograms showing quantification of GluN1 and adaptin β2 association after glycine stimulation with or without L689560 (n = 4). Data are normalized to ECS group and presented as mean percent of ECS control (± s.e.m). **B**, NMDAR internalization measured by cell ELISA assay. Cultures (n = 5) were pre-treated with glycine (100 μM) plus APV (100 μM) with or without L689560 (1μM) followed by NMDA (50 μM) plus glycine (1μM). * indicates p <0.05, ** indicates p < 0.01 compared to L689560-treated group.

To determine whether the effects of glycine are dependent upon the site occupied by glycine when it acts as a co-agonist for NMDAR channel gating, we tested the glycine site antagonist L689560 [22,23]. We found that L689560 had no effect on the basal association of GluN1 and adaptin β2 (Figure 3A). However, application of L689560 with glycine (100 μM) prevented the enhancement of GluN1 co-immunoprecipitation with anti- adaptin β2 (Figure 3A). Additionally, applying L689560 together with glycine (100 μM) prevented the decrease in cell-surface NMDARs evoked by subsequent treatment with NMDA (50 μM) plus glycine (1 μM) (Figure 3B). The effects of L689560 to block the glycine-enhanced AP-2-NMDAR association and the glycine-stimulated reduction in cell-surface NMDARs were observed with GluN1/GluN2A and with GluN1/GluN2B receptors (Figure 3A and B). Thus, the effect of L689560 on recombinant NMDARs matched its effects on native NMDARs in neurons [10].

**Figure 4:**
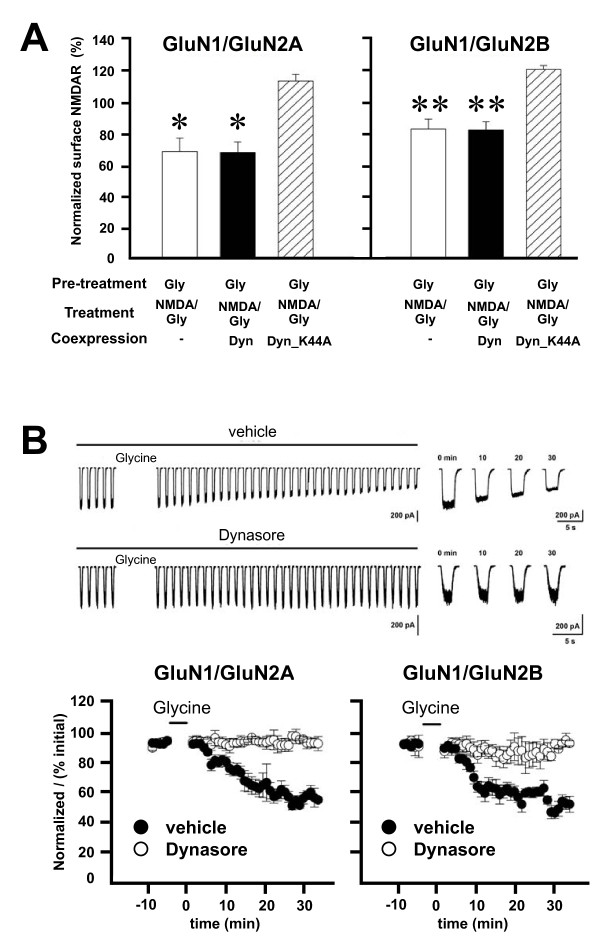
**Glycine primed NMDAR internalization and depression of NMDAR mediated currents require dynamin. A**, Co-expression of mutant dynamin K44A (Dyn_K44A) but not wild-type dynamin blocks glycine primed depletion of surface NMDA receptors (n = 4). * p <0.05, **p <0.01, paired t-test. **B**, Representative traces show responses to the test applications recorded from cells expressing GluN1/GluN2A that were treated with vehicle (DMSO, upper) or treatment (Dynasore, lower). Glycine (100 μM) was applied for 5 min where indicated. In the expanded traces on the right, time indicates time after glycine treatment. The graphs show normalized NMDA-evoked peak currents from recordings with intracellular administration of vehicle or dynamin inhibitor dynasore (80 μM) before and after glycine conditioning (indicated by the bar above each graph) for cells transfected with GluN1/GluN2A (n = 4 cells; left panel) or with GluN1/GluN2B (n = 6 cells; right panel).

Glycine-primed internalization of native NMDARs and depression of neuronal NMDAR currents is prevented by blocking dynamin dependent endocytosis [10]. We therefore examined the effects of dynamin inhibitors on glycine priming and internalization of recombinant NMDARs. First, we used a dominant negative form of dynamin 2 (dynamin2-K44A) [24], which was co-expressed together with recombinant NMDARs. We found that expressing dynamin2-K44A prevented the glycine-induced decrease of cell-surface levels of GluN1/GluN2A and GluN1/GluN2B receptors (Figure 4A). By contrast, expressing wild-type dynamin 2 had no effect on the glycine-primed reduction of cell surface NMDARs. Second, we intracellularly administered dynasore, a non-competitive inhibitor of dynamin 1 and dynamin 2 [25,26], during whole cell recordings. We found that during recordings with dynasore, currents evoked from GluN1/GluN2A or GluN1/GluN2B receptors did not decline after glycine treatment (Figure 4B). By contrast, in vehicle-control cells glycine induced a progressive reduction in NMDA-evoked currents (Figure 4B).

Collectively, these results show that wild-type recombinant NMDARs expressed in HEK293 cells are subject to glycine-primed internalization that is dynamin-dependent. Glycine primes internalization when the receptors are comprised of either GluN1/GluN2A or GluN1/GluN2B subunits. Thus, the characteristics of the glycine-primed internalization of the recombinant receptors fully recapitulate those of glycine-primed internalization of native NMDARs in neurons.

### GluN1 mutant receptors that lack glycine priming

Having established that glycine-primed internalization was recapitulated with recombinant NMDARs, we mutated residues in the ligand-binding domain of GluN1 to test the hypothesis that glycine priming depends upon glycine binding to this subunit. We first used a GluN1 mutant carrying four amino acid substitutions, N710R, Y711R, E712A, A714L, which impaired but did not abolish gating of NMDARs containing this GluN1 mutation [27,28]. We found that NMDARs with this quadruple GluN1 mutation, which we refer to as the RRAL mutant, were expressed at levels comparable to those of wild-type GluN1 when co-transfected with GluN2B, but there was no detectable expression if co-transfected with GluN2A (data not shown). Therefore, we tested glycine priming only with mutant GluN1/GluN2B receptors.

**Figure 5:**
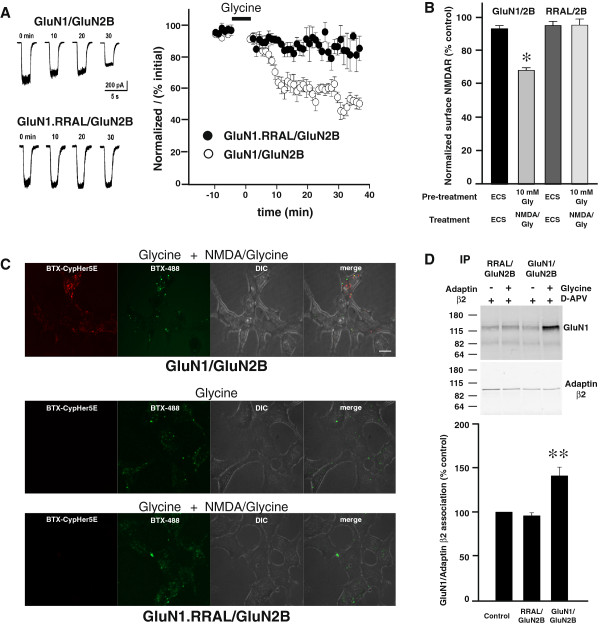
**Mutant GluN1.RAAL/GluN2B receptors did not show glycine priming. A**, *Left*, Traces show individual responses to the test applications recorded from cells expressing GluN1/GluN2B (upper) or GluN1.RRAL/GluN2B (lower). Times indicates time after glycine treatment (10 mM, 5 min); t = 0 is the first response after glycine treatment. *Right*, Normalized NMDA currents (I/I0) vs time from cells expressing GluN1/GluN2B (n = 4 cells) or GluN1.RRAL/GluN2B (n = 9 cells). Glycine (10 mM, 5 min) was applied during the period indicated by the bar above the graph. **B**, NMDAR internalization as quantified by cell ELISA assay using HEK293 cells expressing GluN1/GluN2B or GluN1.RRAL/GluN2B receptors. Cultures (n = 6) were pre-treated with ECS or ECS containing glycine (10mM) plus APV (100 μM) followed by ECS or with NMDA (50 μM) plus glycine (100 μM). * indicates p < 0.05 compared with ECS control. **C**, Cells expressing BBS-GluN1.RRAL/GluN2B showed minimal CypHer5E fluorescence after treatment of NMDA (50 μM) plus glycine (100 μM) with 10 mM glycine plus 100 μM APV pre-treatment. Scale bar = 10 μm. **D**, *Top*, Representative Western blot of co-immunoprecipitation of NMDARs with adaptin β2 from cell lysate treated with glycine (10 mM) plus APV (100 μM) or APV (100 μM) alone. *Bottom*, Quantification was summarized in the histogram showing mean GluN1-adaptin β2 association from HEK293 cells expressing GluN1/GluN2B and GluN1.RRAL/GluN2B receptors after glycine treatment (n = 10). Data are presented as mean percent (± s.e.m.) of APV treated group. Data were normalized to the amount of adaptin β2 immunoprecipitated. ** indicates p < 0.01 compared to control.

We investigated GluN1.RRAL /GluN2B using the four approaches established for wild-type receptors. Consistent with the reported reduction in potency of glycine with RRAL mutant receptors [27,28], applying NMDA (50 μM) and glycine (1 μM) evoked no currents with GluN1.RRAL/GluN2B receptors (not illustrated). However, stimulating with test applications of NMDA (50 μM) plus glycine (100 μM) evoked currents that were stable for at least 40 min (Figure 5A), demonstrating that gating of the mutant receptors is evoked by increasing glycine concentration in the test applications. It was conceivable that the potency of glycine for priming NMDARs might not have been altered in the RRAL mutant. Therefore, we exposed cells expressing the mutant NMDARs to glycine (100 μM) for 5 min and found that there was no subsequent change in the amplitude of the currents evoked by the test applications (data not shown). Thus, the glycine stimulation that primed reduction in current amplitude of wild-type NMDARs had no effect on the GluN1.RRAL/GluN2B mutant.

Because glycine potency for NMDAR gating is reduced in RRAL receptors [28], we examined the effect of treating the mutant receptors with glycine at concentrations in excess of that needed to compensate for the reduction in gating potency. RRAL receptors show a 330-fold reduction in glycine potency for evoking NMDAR currents [28], and therefore we tested glycine concentrations in excess of 330 times the EC_50_ for priming wild-type NMDARs [10]. We found that mutant receptors exposed to glycine at 10 mM showed no subsequent decline in currents evoked by test applications, rather the currents were stable for up to 30 min (Figure 5A). To investigate whether increasing glycine concentration might, paradoxically, prevent the decline in NMDAR currents with wild-type receptors, we exposed cells expressing GluN1/GluN2B to high glycine (10 mM). After this high glycine treatment the amplitude of the test currents declined NMDAR currents to approximately 50% of that before glycine treatment (Figure 5A). Thus, we found no evidence for glycine-primed reduction of NMDAR currents of GluN1.RRAL/GluN2B receptors even when the glycine concentration was increased to compensate for the reduction in gating potency for glycine.

We therefore investigated whether there was a corresponding lack of glycine-primed internalization of the RRAL mutant receptors. Using cell ELISA approach we found that pretreating with glycine (10 mM) followed by treatment with NMDA (50 μM) plus glycine (100 μM) caused no change in cell-surface levels of the mutant receptors (Figure 5B). By contrast, GluN1/GluN2B cell-surface level was significantly decreased to 73 ± 3% (p < 0.05) of ECS control. Moreover, we generated and tested GluN1.RRAL/GluN2B mutant receptors tagged with the BTX-binding sequence at the N-terminus. In contrast to the robust receptor endocytosis observed with wild-type BBS-tagged GluN1/GluN2B receptors, glycine (10 mM) pre-treatment followed by treatment with NMDA (50 μM) plus glycine (100 μM) produced no detectable CypHer5E fluorescent signal in cells expressing BBS-tagged GluN1.RRAL/GluN2B mutant receptors (Figure 5C). However, there was robust cell-surface expression of the mutant receptors as shown by the BTX-AF488 fluorescence signal (Figure 5C). Thus, we conclude from these findings that NMDARs containing the RRAL GluN1 mutation fail to show glycine-primed internalization.

To determine whether the lack of glycine-primed internalization of the mutant receptors may have been due to lack of priming by glycine, as opposed to lack of internalization *per se* of primed receptors, we investigated whether glycine stimulation recruits AP-2 to the mutant receptors. Basal association of adaptin β2 with GluN1.RRAL/GluN2B was comparable to that of wild-type NMDARs. However, glycine (10 mM) did not alter the amount of GluN1.RRAL that co-immunoprecipitated with anti-adaptin β2 (Figure 5D). The association of wild-type receptors with adaptin β2 significantly increased upon treatment with glycine (10 mM) (Figure 5D). As glycine does not enhance the association between AP-2 and the mutant NMDARs we conclude that GluN1.RRAL/GluN2B receptors lack glycine priming.

### GluN1 A714L mutation abolishes glycine priming

**Figure 6:**
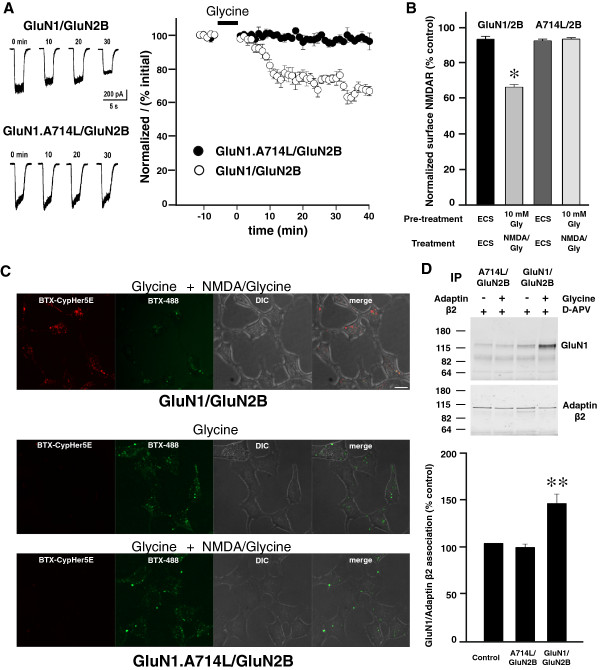
**A714L mutation in GluN1 prevents glycine primed NMDAR internalization. A**, *Left*, Traces show responses to the test applications recorded from cells expressing GluN1/GluN2B (upper) or GluN1.A714L/GluN2B (lower). Times indicates time after glycine treatment (10 mM, 5 min); t = 0 is the first response after glycine treatment. *Right*, Normalized NMDA currents (I/I0) vs time of the experiment from cells expressing GluN1/GluN2B (n = 4 cells) or GluN1.A714L/GluN2B (n = 3 cells) receptors. Glycine (10 mM, 5 min) was applied during the period indicated by the bar above the graph. **B**, NMDAR internalization measured by cell ELISA assay using HEK293 cells expressing GluN1.A714L/GluN2B receptors (n = 6). * indicates p < 0.05 compared to ECS control. **C**, Cells expressing BBS-GluN1.A714L/GluN2B showed no internalization as measured by CypHer5E fluorescence in cells. Scale bar = 10 μm **D**, *Top*, Representative Western blot of co-immunoprecipitation of NMDARs with adaptin β2 from cell lysate treated with glycine (10 mM) plus APV (100 μM) or APV (100 μM) alone. *Bottom*, Quantification is summarized in the histogram showing mean GluN1-adaptin β2 association from HEK293 cells expressing GluN1/GluN2B and GluN1.A714L/GluN2B receptors after glycine treatment (n = 10). Data are presented as mean percent (± s.e.m.) of APV treated group. Data were normalized to amount of adaptin β2 immunoprecipitated. ** indicates p < 0.01 compared to control.

Of the four amino acid changes in the RRAL mutant, only A714L impairs glycine potency as a single point mutation [28]. Therefore, we investigated the effect of alanine to leucine mutation at residue 714 (GluN1.A714L) on glycine-primed internalization of NMDARs. GluN1.A714L/GluN2B receptors formed functional NMDARs as illustrated by the currents evoked by applying NMDA (50 μM) plus glycine (100 μM; Figure 6A). We found that treating GluN1.A714L/GluN2B receptors with glycine, at concentrations up to 10 mM, had no effect when investigated with any of the four approaches: i) NMDA-evoked currents were stable after glycine (10 mM) treatment (Figure 6A), ii) cell-surface GluN1.A714L/GluN2B receptor levels did not change with glycine pre-treatment (10 mM) followed by activation with NMDA (50 μM) plus glycine (100 μM; Figure 6B), iii) GluN1.A714L/GluN2B receptors did not internalize after glycine pre-treatment (10 mM) followed by receptor activation with NMDA (50 μM) plus glycine (100 μM; Figure 6C), and iv) association of AP-2 with the GluN1.A714L/GluN2B receptors did not change with glycine (10 mM) treatment (Figure 6D).

Thus, the single mutation of alanine to leucine at 714 in GluN1 was sufficient to prevent all of the indicia of glycine-primed internalization. The potency of glycine at GluN1.A714L receptors has been shown to be reduced only 62-fold compared with that of wild-type receptors [28]. Thus, A714L mutation abolished glycine priming even though glycine concentration was increased far more than needed to compensate for the reduced glycine potency for gating the GluN1.A714L mutant receptor.

## Discussion

In this study we found that with wild-type NMDARs comprised of GluN1/GluN2A or GluN1/GluN2B: i) glycine primed an approximate 50% reduction in NMDA-evoked currents, ii) glycine pre-treatment induced a dramatic reduction in NMDAR cell-surface levels upon subsequent NMDAR activation, iii) glycine pre-treatment, with subsequent NMDAR activation, provoked robust NMDAR internalization into an acidic intracellular compartment; iv) glycine recruited AP-2 to the NMDAR complex. These effects of glycine were blocked by a glycine-site antagonist or by disrupting dynamin function. Thus, like native NMDARs, wild-type recombinant NMDARs undergo homologous glycine-primed internalization that is dynamin-dependent. The glycine priming process was observed with NMDARs comprised of either GluN1/GluN2A or GluN1/GluN2B and thus priming is not dependent upon which of the two GluN2 subunits is partnered with GluN1.

In contrast to wild-type NMDARs, the mutant NMDARs examined showed no signs of glycine priming or of glycine-primed internalization. Specifically, with NMDARs formed of GluN1.RRAL/GluN2B or of GluN1.A714L/GluN2B: i) glycine application did not cause a change in NMDA-evoked currents; ii) NMDAR cell-surface levels were unchanged by glycine pre-treatment with subsequent NMDAR activation; iii) glycine pre-treatment led to no NMDAR internalization upon subsequent NMDAR activation; iv) AP-2 was not recruited to the NMDAR complex by applying glycine. Both of the mutant GluN1 subunits share conversion of alanine at position 714 to leucine, and even the mutation of this residue alone prevented glycine priming. Thus, our findings demonstrate that the single amino acid in GluN1, A714, is critical for glycine priming of NMDARs.

This critical residue at position 714 is within the ligand-binding domain of GluN1 which is comprised of two polypeptide segments, S1 and S2 [29]. The S1S2 segments form a bilobed structure. Crystallographic analysis of GluN1 S1S2 has revealed that, like other ionotropic glutamate receptors, unliganded apo GluN1 is in an open conformation where S1 and S2 are apart, like an open clamshell. Binding of glycine stabilizes a closed conformation where S1 and S2 are in apposition like a closed clamshell. This closed conformation of S1S2 of GluN1, when occurring together with agonist binding to the glutamate site in S1S2 of GluN2, induces a cascade of conformational changes in the receptor complex which ultimately leads to a conformational state where the channel pore is open [30-37]. Lack of glycine-induced recruitment of AP-2 in receptors carrying the A714L mutation is strong evidence that S1S2 closure couples not only to channel pore opening but also to additional conformational changes that allow AP-2 binding. As AP-2 binds to the intracellular region of the NMDAR complexes, conformational changes induced by S1S2 closure must be transduced across the cell membrane [6].

A714 does not coordinate directly with bound glycine [29], and therefore, reduction in glycine potency of NMDARs containing the GluN1 A714L mutation may be attributed to destabilization of the glycine-bound closed conformation of GluN1 S1S2 causing inefficient coupling to channel pore opening. The open conformational state of the A714L mutant receptor complex is nevertheless achieved as shown by the inward currents evoked by applying NMDA plus glycine. But even at concentrations far in excess of those needed to compensate for changes in the potency for gating, glycine failed to recruit AP-2 to the mutant NMDARs. This lack of glycine-induced recruitment of AP-2 to the mutant receptor complexes demonstrates clear molecular dissociation of NMDAR priming from gating. The most parsimonious explanation for these findings is that destabilization of the closed S1S2 of GluN1 A714L, which only partially reduces coupling to channel opening, eliminates coupling to the conformational changes necessary for recruiting AP-2. If the NMDAR complex cannot undergo the conformational changes needed to recruit adapter proteins, as with the A714L mutants, then the remaining endocytic machinery cannot be assembled and endocytosis is prevented.

Recruitment of AP-2 induced by stimulating with glycine is prevented by the glycine-site antagonist L689560 and, as well, L689560 alone did not cause AP-2 recruitment. Binding of antagonists to S1S2 of ionotropic glutamate receptors is believed to lead to a partially closed state of the S1S2 which is unable to couple to gating [29]. Our findings indicate that the conformation induced by binding of glycine-site antagonists is not a conformation capable to recruit the core endocytic adaptor. Moreover, binding of glutamate-site antagonists prevented, and did not cause, NMDAR internalization indicating that the remaining molecular machinery needed for endocytosis was not subsequently assembled by antagonist-bound NMDARs. In contrast, the non-competitive antagonist MK-801 does not prevent glycine-primed internalization of NMDARs (Nong et al., unpublished observation; see also Vissel et al. 2001), indicating that ion flux through NMDARs is not required for assembling the endocytic machinery. Blockade of priming and endocytosis of NMDARs by glycine and glutamate site antagonists, respectively, contrasts with homologous internalization of AMPA receptors where antagonists as well as agonists cause receptor internalization [38,39]. Thus, consequences of the conformational changes induced by antagonist binding NMDARs are distinct from those of AMPARs and there is no general rule for effects of antagonists on homologous endocytosis of ionotropic glutamate receptors.

The consequences of glycine-site occupancy reflect differential coupling to two distinct effector outcomes –channel pore opening or recruitment of endocytic adaptors. Coupling of agonist occupancy to multiple effectors is well known for other cell-surface receptors such as G-protein coupled receptors (GPCRs) [40]. For GPCRs, a single type of receptor may couple to a large number of distinct effectors, with the degree of coupling to specific sets of effectors often determined by the ligand that activates the receptor [41]. Evidence from pharmacological and structural studies indicates that GPCRs adopt multiple agonist-bound conformations which are able to recruit different downstream binding partners and that stabilization of different active conformations of the receptors engages distinct subsets of effectors [41,42]. Thus, the conformational differences in NMDARs induced by glycine that we infer lead to channel gating versus to priming/endocytosis are analogous to the conformational differences that underlie structure-biased effector coupling with GPCRs. With GPCRs there is increasing structural information about the intracellular regions of the receptors and their binding to different effector proteins [43]. We anticipate that such structural information about NMDARs will ultimately provide the atomic level detail needed to understand the channel gating and priming effects of GluN1 binding of glycine.

## Conclusions

In summary, we find that mutating alanine to leucine at position 714 of GluN1, either alone or in tandem with other point mutations, prevented glycine priming of NMDARs. This critical amino acid is in the ligand-binding region of GluN1, indicating that binding of glycine to this NMDAR subunit is essential for priming the receptors. Importantly, NMDARs with the A714L GluN1 mutation are functional channels when activated with the co-agonists NMDA and glycine. Thus, our findings demonstrate that the molecular determinants in GluN1 for priming NMDARs by glycine are separable from those for gating NMDARs by glycine acting as a co-agonist.

## Methods

### Molecular biology

Mammalian expression vectors encoding wild-type rat GluN1-1a, GluN2A, and GluN2B cDNAs have been previously described [10]. The A714L mutation [28] and the N710R Y711R E712A A714L (RRAL) mutations [27] were introduced using the QuickChange site directed mutagenesis kit (Stratagene, La Jolla, California). All constructs were verified by DNA sequencing. Wild-type and dominant-negative (K44A) mutant forms of dynamin2 were generously provided by S. E. Egan [24].

### Cell culture and transfection

Human embryonic kidney cell line (HEK293) cells (3 × 10^4^ cells/cm^2^) were plated onto 6-well culture dishes coated with poly-D-lysine. HEK293 cells were cultured with Dulbecco’s Modified Eagles Media (DMEM) (Invitrogen, Burlington, ON, Canada) supplemented with 10% fetal bovine serum (Invitrogen) and 1% penicillin-streptomycin (Wisent, St. Bruno, QC) ); 37°C, 5% CO_2_. For electrophysiological recordings in HEK293 cells, low density cultures were plated 24 h before transfection on poly D-lysine-coated glass coverslips. FuGene HD (Promega BioSciences,LLC.: Sand Luis Obispo, CA, USA) transfections always included GluN1-1a; a GluN2 construct, either 2A or 2B; and PSD-95 at a DNA ratio of 1:4:0.5. For electrophysiological recordings a plasmid containing enhanced green fluorescent protein (eGFP) was also included in the transfection at a ratio of 0.5 with respect to GluN1. After transfection, cells were maintained in DMEM supplemented with 10% fetal bovine serum and D-APV (500 μM; Tocris, Minneapolis, MN, USA) for 48 hrs before experiments.

### Co-immunoprecipitation assay

HEK293 cells transfected with wild-type or mutant constructs were treated for 5 min with extracellular solution (ECS) (pH 7.35, 330 mOsm; 140 mM NaCl, 1.3 mM CaCl2, 5 mM KCl, 25 mM HEPES, 33 mM glucose) supplemented with glycine site agonists and/or antagonists, or other reagents, as indicated. Cells were homogenized in ice cold lysis buffer (50 mM Tris–HCl (pH 8.0), 150 mM NaCl, 2 mM EDTA, 0.1% SDS, 1% NP-40, 0.5% sodium deoxycholate, Complete Protease Inhibitor Cocktail Tablets (Roche, Indianapolis, IN, USA)). Insoluble material was removed by centrifugation at 14,000 g for 20 min at 4°C. Cell lysates were incubated overnight with 2 mg of anti-AP-2 adaptin β2 (BD Biosciences). Immune complexes were isolated by addition of 20 μl of mouse protein G-Sepharose beads (GE Healthcare, Sweden), followed by incubation for 1–2 h at 4°C. Immunoprecipitates were then washed four times with lysis buffer, resuspended in laemmli sample buffer, and boiled for 5 min. The proteins were separated by SDS–polyacrylamide gel electrophoresis (PAGE), and transferred to a nitrocellulose membrane. Nitrocellulose membranes were immunoblotted with anti-GluN1 or with anti-adaptin β2 primary antibodies, and their respective secondary antibodies conjugated to IR800 and IR700 (Rockland). Antibody signals were quantified using the LICOR imaging system (LI-COR Biosciences, Lincoln, NE, USA). Serial dilutions were used to confirm that under these experimental conditions signal intensities for GluN1 or adaptin β2 were linear over a 50-fold range. We note that immunoprecipitating with a non-specific IgG caused no detectable precipitation of GluN1 or adaptin β2.

### Colorimetric cell enzyme-linked immunosorbent assay (ELISA)

Assays were carried out as previously described [10]. Briefly, HEK293 cells transfected with wild-type or mutant NMDARs were cultured in 12-well plates (approximately 2.5 × 10^5^ cells per well). After removing the media, HEK cells were covered in ECS and cooled to 4°C to inhibit membrane trafficking. To pre-label cell surface NMDA receptors, the cells were incubated for 1 hr at 4°C with an anti-GluN1 antibody against the extracellular domain of GluN1 (BD biosciences; 2 μg ml^-1^). After treatment with vehicle or ligands, HEK293 cells were fixed with 4% paraformaldehyde in phosphate buffered saline (PBS, Wisent) without detergents to prevent permeabilization. After washing, cells were incubated for 1 hr at room temperature with a horseradish peroxidase-conjugated secondary antibody (1:1,000, Amersham). The color reaction was produced by adding chromagenic substrate (OPD; Sigma, St Louis, Missouri, USA) and stopped with 0.2 volume of 3N HCl. The optical density of the supernatant (1 ml) was read on a spectrophotometer at 492 nm. The levels of cell surface expression of NMDARs were presented as a ratio of colorimetric readings measured on cells not subject to the 15 min incubation at 37°C.

### Generation of α-bungarotoxin-binding-site tagged GluN1

A 13-aa α-bungarotoxin (BTX)-binding site (BBS) sequence [CTAGCTGGAGATACTACGAGAGCTCCCTGGAGCCCTACCCTGACA (sense) and CTAGTGTCAGGGTAGGGCTCCAGGGAGCTCTCGTAGTATCTCCAT (antisense) [21]] was subcloned into a Hind III site introduced downstream of the signal peptide in the GluN1-1a subunit, referred herein as BBS-GluN1-1a, and subcloned into pAEMXT-ACPwt (Covalys).

### CypHer5E mono NHS ester conjugation to BTX

CypHer5E N-hydroxysuccinimidyl ester (GE Healthcare, Buckinghamshire, UK) was conjugated to unlabeled BTX (Molecular Probes, Invitrogen) according to the manufacturer’s instructions. Briefly, BTX was diluted to 1 mg/ml in PBS and 0.5 M sodium carbonate buffer, pH 8.3, and then incubated with 50-fold molar excess of CypHer5E NHS for 1 hr at room temperature in the dark. The CypHer5E-conjugated BTX (BTX-CypHer5E) was separated from free CypHer5E by dialysis in PBS overnight at room temperature. The molar concentration of antibody and dye in the final sample was then calculated by measuring the absorbance of the labeled BTX at 280 and 500 nm. The mean number of dye molecules coupled to the BTX was then determined. The BTX-CypHer5E was diluted to 0.5 mg/mL with PBS containing 0.1% BSA and stored frozen at −20°C.

### Optical recording of internalization of NMDARs in live cells using BTX-CypHer5E

We used CypHer5E, a pH-sensitive cyanine dye derivative, excited at 633 nm and only fluorescent in acidic environments, to track agonist-induced NMDAR endocytosis. Surface BBS-NMDARs were labeled with 3 μg/ml BTX-CypHer5E at 4°C for 30 min, washed and pre-treated at 37°C with control ECS or 100 μM glycine for 5 min. The labeling was sufficient to allow tracking of NMDARs without saturating all the BBS-NMDARs. Live cells were then treated with control ECS or NMDA (50 μM) plus glycine (1 μM) for 10 min. After washing with cold ECS, cells were incubated with Alexa Fluor®488-conjugated BTX (BTX-AF488, Molecular Probes; 10 mM) at 18°C for 20 min. Cells were washed to remove unbound BTX-AF488 and then imaged using confocal microscopy (Olympus IX81). Images were collected by a Hamamatsu Back-Thinned EM-CCD camera using the Volocity software (Perkin Elmer). Final processing was performed with Adobe Photoshop CS5 without changing the original resolution and color depth.

### Whole-cell recording

Whole-cell patch clamp recordings were generated from HEK293 cells expressing recombinant wild-type or mutant NMDARs together with GFP. Cells on cover slips were transferred to a recording chamber and continually perfused (approximately 1 ml min^-1^) in ECS (in mM): NaCl, 140; KCl, 5.4; CaCl_2_, 1.3; Hepes, 25 and D-glucose, 33; Glycine, 0.001 (pH adjusted to 7.4 with NaOH). Cells were visualized on an inverted microscope (Nikon) equipped with epi-fluorescence and a GFP filter set. Patch pipettes were made from borosilicate glass (World Precision Instruments) using a Brown-Flaming horizontal puller (Model P-97, Sutter Instruments Co.) and were fire-polished (MF-830 Microforge, Narishige). Micropipettes had a resistance of 5–7 MΩ, formed gigaseals between 2 and 12 GΩ and were filled with intracellular recording solution (in mM): CsF, 140; BAPTA, 10; Hepes, 10 and MgATP, 2 (pH adjusted to 7.2 with CsOH). Once a gigaseal was formed, the cell was lifted up from the cover slip to allow the ECS to flow to all surfaces of the cell. The cell membrane potential was clamped at −60 mV. NMDAR currents were evoked by test applications (3 s duration) of NMDA (50 μM) and glycine (1 μM) at 60 sec intervals with a SF-77B Perfusion Fast-Step system (Warner Instruments). Applications of NMDA/glycine were made for 5–10 min in order to establish a stable NMDAR current baseline. Current traces were filtered at 2 kHz, digitized at 10 kHz and stored on a PC for later analysis. Capacitive transients were minimized by analogue means (in Figures, residual transients have been truncated for illustrative purposes). Current amplitudes were measured at maximum inward peak for each NMDA application. All analyses and voltage protocols were performed using an Axopatch 1D amplifier in combination with a Digidata 1200A interface and pCLAMP 9.0 software (Molecular Devices). All recordings were made at room temperature (20–22°C). NMDA-evoked current data are presented as percentage of the peak mean current (I) normalized to the initial response (I0). All data are presented as means ± s.e.m. Where indicated, the dynamin inhibitor, dynasore (80 μM) [25], was applied through the patch pipette. Dynasore was dissolved in DMSO, final DMSO concentration (0.2%). Once whole-cell configuration was achieved, we allowed 10–15 min for diffusion to the cell cytoplasm and then started recording NMDA-evoked currents. Thus, dynasore was present before, during and after glycine priming. Control experiments were performed in with DMSO alone (0.2%) applied through the patch pipette.

### Glycine priming protocol

For glycine priming experiments, we made a 5 min application of glycine and D-APV (100 μM) with or without glycine site antagonist L689560 (1 μM) [16,22,23] in ECS. The glycine concentration was normally 100 μM. But in experiments with mutant NMDARs glycine was used, where indicated, at concentration of 10 mM. Note that D-APV was included with all glycine priming treatments in all types of experiment in order to avoid activating NMDAR channel gating. Afterwards, the glycine priming solution was washed away for 1 min using control ECS, before re-probing NMDAR activity with the test NMDA plus glycine applications every 60 s.

### Statistical analysis

Statistical comparisons were made using Student’s *t*-test or a non-parametric test (Mann–Whitney), as appropriate; differences with p < 0.05 were considered statistically significant. SigmaPlot v11.0 software was used for graphical presentation. Results are presented as mean ± SEM.

## Competing interests

The authors declare that they have no competing interests.

## Authors’ contributions

LH, VAC and JC performed experiments and conducted data analysis. LH and MWS drafted and edited the manuscripts. All authors read and approved the final manuscript.

## Supplementary Material

Additional file 1: Figure S1 Treatment of HEK293 cells with 100 mM glycine for 5min did not change level of association between GluN1 and Adaptin β2 protein. Click here for file
