# Supplementary material for: Identification of a single amino acid in GluN1 that is critical for glycine-primed internalization of NMDA receptors
Source: Mol Brain. 2013 Aug 13;6:36. doi: 10.1186/1756-6606-6-36 (PMC3846451; doi:10.1186/1756-6606-6-36)
Supplement: Additional file 1: Figure S1 — Treatment of HEK293 cells with 100 mM glycine for 5min did not change level of association between GluN1 and Adaptin β2 protein. [file 1756-6606-6-36-S1.pdf]

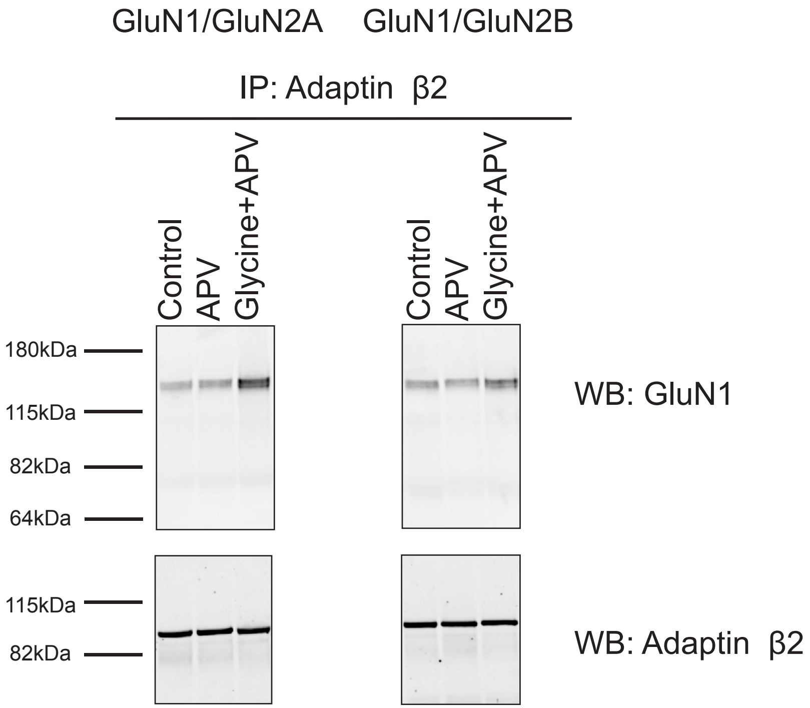

Supplementary figure 1. Treatment of HEK293 cells with 100 μM glycine for 5min did not change level of association between GluN1 and Adaptin β2 protein.
